# Thyroid Surgery for Elderly Patients: Are They at Increased Operative Risks?

**DOI:** 10.1155/2012/946276

**Published:** 2012-08-16

**Authors:** Sze-How Ng, Kai-Pun Wong, Brian Hung-Hin Lang

**Affiliations:** ^1^Breast and Endocrine Unit, Department of Surgery, Kuala Lumpur Hospital, 50586 Kuala Lumpur, Malaysia; ^2^Division of Endocrine Surgery, Department of Surgery, The University of Hong Kong, Queen Mary Hospital, Pokfulam, Hong Kong

## Abstract

An increasing elderly population, a rising incidence of differentiated thyroid carcinoma (DTC), and a rising incidence of benign nodular disease with age are all contributing to a rise in thyroid operations for the elderly. Literature review on the outcome and safety of thyroid surgery in elderly patients has been filled with conflicting results and this subject remains controversial. Although most single-institution studies conducted by high-volume surgeons did not find significant differences of complication rates in elderly when compared with younger cohorts, they often lacked the power necessary to identify subtle differences and suffered from various selection and referral biases. Recent evidence from large population-based studies concluded that thyroid surgery in the elderly was associated with higher complication rates. One of the major contributing factors for the increased complication rate was because most elderly patients suffered from many preexisting comorbidities. Therefore, elderly patients who have abnormal thyroid findings should complete a thorough preoperative workup and better postoperative care after undergoing any thyroid surgery. Furthermore, these high-risk patients would benefit if they could be referred to high-volume, specialized surgical units early. In this systemic review, we aimed to evaluate different issues and controversies in thyroidectomy for elderly patients.

## 1. Introduction

Both benign and malignant thyroid diseases occur commonly in the elderly population and this is partly related to the fact that the incidence of thyroid nodules increase with age [1]. It is clear that our general population is aging rapidly and the proportion of the elderly has increased by 90% over the last 30 years. It is projected that by the year 2020, the proportion of patients aged ≥65 years old would reach 20% from the 12.4% [2]. Since thyroid surgery remains one of the most commonly performed operations worldwide, thyroid surgery in the elderly has become an important topic in endocrine surgery [3–6]. In general, the common surgical indications such as thyroid cancer, multinodular goiter (MNG), and hyperthyroidism are similar between the young and elderly patients [7, 8]. However, since it is generally perceived that the elderly may have increased operative risks because of the presence of increasing number of comorbidities, a more conservative or nonoperative approach is often adopted [3]. As a result, those patients who really undergo thyroid surgery generally would normally have a much “stronger” or more urgent indication, such as those with more severe symptoms from their thyroid disease (e.g., compressive symptoms) or those with suspected or proven malignancy, than their younger counterparts. However, these more urgent indications also generally pose increased operative risk and so age per se may not account for the increased operative risk [6, 7]. Furthermore, over the last 10 years, major advances have occurred in general and endocrine surgery, anesthesia, perioperative care, instrumentation, and technology and they tend to reduce the overall operative risk, allowing greater proportion of elderly patients to undergo elective surgery [9]. To our knowledge, information on the elderly population undergoing elective thyroid operation remains scare. Furthermore, there is no universally agreed definition on the term “elderly”. The cutoff age for “elderly” may vary from 60–80 years or older [3–6, 10, 11]. The current literature on the outcome and safety of thyroidectomy in elderly patients has been filled with conflicting results and the topic remains controversial. Most single-institution studies concluded that thyroidectomy in the elderly was not associated with increased complications when compared with their younger counterparts [3, 5, 10, 12, 13]. However, several recent studies including two large population-based series [4, 6] concluded that thyroidectomy in the elderly were associated with higher risk for complications. In this review, we aimed to evaluate both safety and surgical outcome on thyroid surgery in elderly patients. At the same time, it would be important to examine the actual cost and impact on quality of life in the elderly if elective thyroid operation is decided.

## 2. Demographic and Clinical Characteristics

### 2.1. Age and Gender

As mentioned earlier, there has not been any clear definition for the elderly or geriatric patient in the medical literature. In the past, authors used different definitions or age groups to define elderly patients [3–6, 10, 11]. Some studies defined any patients aged 65–79 years old as “elderly” and those aged 80 years and older as “superelderly” [4, 14]. Bliss et al. defined patients 75 or older as elderly [3] and some even described patients as young as 65 years as “elderly” [10]. Lang and Lo referred to patients 70 years or older as elderly in a retrospective analysis on multinodular patients [5]. Mekel et al described patients 80 years or older as octogenarians or elderly group [15]. As a result of this difference in definition, the actual proportion of elderly patients undergoing elective thyroid surgery ranged between 2.5% to 21.2%, depending on patient selection and type of referral (Table 1) [3–6, 10, 15, 16]. In terms of sex ratio, most reported series found that their cohort was predominantly female with a female: male ratio of 4 : 1 [3, 4, 15]. This is mostly because thyroid diseases mostly appear in the female sex and also because the females tend to live longer and stay healthier for longer than the males and so they are more likely to be fit for surgery.

### 2.2. Presentation and Surgical Indications

In the literature review, the main indications for surgery in elderly patients were suspected or verified malignancy, mechanical or compression symptoms, and followed by substernal or retrosternal goiter (Table 1) [3, 5, 15, 16]. Bliss et al. reported that the main indications for surgery in elderly were airway compression and risk of malignancy [3]. Additionally, the percentage of retrosternal goiter causing compression was also significant higher in elder as compared to younger group [3]. These results were consistent with Passler et al. who demonstrated that suspected malignancy and mechanical symptoms were significant higher in elderly patients whereas benign goiters were the main indication for younger patients [16]. Lang and Lo evaluated 279 patients undergoing total thyroidectomy for MNG and reported that in addition to retrosternal MNG, toxic MNG was also a common surgical indication [5]. However, it should be pointed out that the prevalence of retrosternal goiter would be significantly higher if these series also included those who opted for non-operative approach (i.e., observation or alternative medicines). In our clinical practice, with increasing use of axial imaging like CT or MRI, it is not uncommon for us to receive referrals for managing elderly patients with incidental finding of retrosternal goiter. Perhaps, a future study could look at the natural course or history of elderly patients with relatively asymptomatic retrosternal goiter and see what proportion of patients without surgery would become symptomatic and what proportion would benefit from early surgery. In our experience, on average, we would see one elderly patient per year requiring emergency admission through the accident and emergency because of acute airway obstruction from a large compressive goiter [17].

It is well known that thyroid cancer tends to behave more aggressively and presents at a more advanced stage in younger patients [3, 18, 19]. In fact, the current TNM has used the age of 45 years old as the cutoff between good and bad risks. As a result, the incidence of local or lymph node recurrence as well as proportion of secondary thyroid surgery tends to be significant higher in elderly group [3, 16]. Matsuyama et al. evaluated 85 patients with thyroid cancers and demonstrated that successful surgery for thyroid cancer increased the survival rate and improved the quality of life of elderly patients [20]. Nevertheless, the decision to perform surgery is not often straightforward as reoperation for persistent or recurrent thyroid cancer is generally associated with increased surgical morbidity and reduced chance of actual cure when measured by stimulated thyroglobulin level alone [21]. Mekel et al. evaluated 332 patients undergoing thyroid surgery and they failed to demonstrate any significant difference between octogenarian and younger patients in terms of preoperative indication for surgery which included benign disease, suspected malignancy, and suspected follicular neoplasm [15].

## 3. Surgical Procedure and Strategy

### 3.1. Comorbidity Indices

In general, patient comorbidity in the literature was assessed either with Charlson comorbidity index [22] or American Society of Anesthesiologists (ASA) class [23]. The Charlson comorbidity index was originally designed to classify prognostic comorbidity in longitudinal study. It has been used in number of studies to stratify patients adjusting for confounding influence of comorbid conditions on outcome and overall survival. Charlson index score was significantly higher in the octogenarians when compared to the younger patients [15]. Sosa et al. demonstrated that the superelderly (i.e., those aged ≥80 years old) had significantly greater comorbidity burden with nearly 36% of patients having 3 or more comorbidities in addition to their thyroid diagnosis [6]. The elderly patients also had higher American Society of Anesthesiologists (ASA) class. An ASA score >3 was found to be an independent risk factor for nonsurgical-related complications after thyroid surgeries but was not associated to increased incidence of myocardial infarction or congestive cardiac failure [4]. The ASA status of patients should be assessed preoperatively by the anesthesiologist, as any evidence of increased risk should result in an altered patient management scheme, including pre-, intra-, and postoperatively. In other words, the ASA should in fact be part of the decision-making process in the surgeon's office before operative intervention.

### 3.2. Types of Procedures

Thyroid surgery remains the treatment of choice for benign and malignant thyroid diseases. Because of longer life expectancy and improved methods of disease detection and treatment, there will be an increased demand for thyroid surgical procedures in the elderly patients population [24]. Bliss et al. reported a higher incidence of secondary procedures on elderly patients and significant difference between the proportion of total and hemithyroidectomies (Table 1). The latter was significantly more common in the younger group than in the elderly groups [3]. Generally, older patients were less likely to undergo total thyroidectomy than younger patients as overall patients had more preoperative comorbidities as reflected by a higher ASA class [4, 6]. In addition, the older thyroidectomy patients appear to have more advanced benign disease as substernal thyroidectomies were required for elderly and superelderly patients as compared to younger group [6].

Thyroid cancers behave generally more aggressive with increasing age [18, 25]. Park et al. evaluated 8899 patients who undergoing thyroidectomy and radioactive iodine (RAI) from the Surveillance, Epidemiology, and End Results (SEER) database in United States and found that older patients aged ≥65 years were more likely to have multiple primary tumours, advanced-staged disease, larger tumour, extrathyroidal extension, and nonpapillary histology [26]. Despite their more aggressive disease, older patients were less likely to undergo near-total/total thyroidectomy, lymphadenectomy, or to receive adjuvant radiation. These trends were pronounced among those aged ≥80 years [26]. However, Matsuyama et al. compared patients with clinicopathological characteristics of Papillary thyroid carcinoma (PTC) between the elderly and young groups and demonstrated that there was no significant difference in the percentage of patients with tumour size ≥4 cm, lymph node metastasis ≥3 cm, and distant metastasis between these two groups [20].

Indications for near-total/total thyroidectomy and RAI in patients with DTC have remained controversial. Because of the generally good prognosis of DTC, prospective randomized trials of treatment with meaningful clinical endpoints, such as tumor recurrence, distant metastasis, or cancer-related death, have been difficult if not impossible to perform, because they would require a prohibitive number of randomized patients and length of followup to achieve sufficient power [27]. Overall, retrospective analyses indicated that total thyroidectomy with RAI and suppressive hormone therapy improved disease-free survival, reduced disease recurrence, eradicated intrathyroid cancer, and facilitated the use of whole-body iodine scans and thyroglobulin surveillance monitoring for recurrence [28, 29]. Therefore, major professional societies, such as the American Association of Clinical Endocrinologists, the American Association of Endocrine Surgeons, the National Comprehensive Cancer Network, and the American Thyroid Association (ATA), have recommended near-total or total thyroidectomy as the initial procedure of choice for DTC and the addition of radioiodine ablation for patients who have functioning remnants in the thyroid bed or distant metastases [26, 30]. In addition, the ATA also suggests that age **>**45 years also may be a criterion for recommending near-total or total thyroidectomy because of higher recurrence rates in this age group [30, 31].

## 4. Complications and Cost

Table 2 shows a summary of the actual monetary cost in surgical series on thyroid surgery in the elderly. However, the cost remains understudied. The risk of thyroidectomy complications in elderly patients remains unclear. It has been suggested that thyroid surgery in the elderly should be restricted to those whose lesions are at higher risk of malignancy, whereas others with little risk of malignancy may be followed up [22]. However, since the standard of care in thyroid surgery has improved, some of the elderly patients are now referred to specialized endocrine surgical units as these units tend to have better overall outcomes and lower complications than less specialized centers [3].  Table 3 shows a summary of the surgical series on thyroid surgery in the elderly.

### 4.1. Surgical-Related Complications

Previous single-institution studies, which looked at complications in elderly patients undergoing thyroid surgery, did not find significant higher complication rates than younger patients [3, 5, 10, 13, 16]. Passler et al. [16] compared the outcomes of 55 patients aged ≥75 years with 683 younger patients and found a rate of 25.5% early postoperative complications in the elderly compared with 22% in the younger group (Table 3). Early postoperative recurrent laryngeal nerve palsy and postoperative hypocalcemia were the most frequent complications in both age groups. In one study, early postoperative recurrent laryngeal nerve palsy was found in 6 (6.3%) of 95 nerves at risk in elderly and 46 (3.9%) of 1172 nerves at risk in younger group. However, these differences were not statistically significant and were consistent with other centers [5, 10]. Although one study also suggested a trend toward higher complications in the elderly, however it was insignificant [16]. There were other studies reporting surgically related complications such as hypoparathyroidism and recurrent laryngeal nerve palsy but found no significant difference between the elderly and young patients [3, 5, 10, 13]. In contrast, two recent large population-based studies reported that thyroid surgery in the elderly was associated with higher complication rates [4, 6]. Sosa et al. evaluated the outcomes after thyroidectomy in patients 65 of age and older in American and they demonstrated that endocrine-specific complications for elderly and superelderly thyroid surgery patients are considerably worse when compared to the young patients [6].

### 4.2. Nonsurgical-Related Complications

Using the American College of Surgeons National Surgical Quality Improvement Program (ACS-NSQIP) data, Grogan et al. found that those aged between 65–79 years old were twice (OD = 2.1, 95% CI: 1.4–3.3) as likely and those aged 80 years or old five times (OD = 4.9, 95% CI: 2.5–9.6) as likely to have a complication compared with younger counterparts [12]. Furthermore, they found that advanced age was a significant risk factor for pulmonary, cardiac, and infectious complications [4]. In addition, they found that the highest rate of complications in the elderly and superelderly groups was for the pulmonary origin. The common non-surgically related complications included congestive cardiac failure, atrial fibrillation, readmission for acute renal failure, urosepsis, and transfusion requirement. Only a single center study reported that the nonsurgically related complications were significant higher in the elderly group (18.9%) as compared to the younger group (5.2%) [15]. Lang and Lo [5] evaluated total thyroidectomy in elderly patients and also demonstrated a slightly higher incidence of overall non-surgical related complications (5.5% versus 0.4%, *P* = 0.21) such as pneumonia in the elderly group (4.3% versus 0.0%, *P* = 0.003) (Table 3) [5]. Passler et al. also reported a higher incidence of respiratory distress (3.6%) in elderly group but it was no significant as compared to the younger group (0.1%) [16]. Nevertheless, it is important to note that in general thyroidectomy is a very safe operation with an overall postoperative complications rate of around 1.3% [4].

### 4.3. Hospital Stay

Sosa et al. reported that average length of stay (LOS) or hospital stay for patients aged 80 years and older was 60% longer than for similar patients aged 65 to 79 years [6]. This finding was especially common in patients 80 years and older who had substantial comorbidities. Lang and Lo evaluated 279 patients who underwent total thyroidectomy for MNG showed that the overall hospital stay for elderly patients (6.4 ± 7.2 days versus 3.7 ± 2.4 days, *P* < 0.001) was statistically and significantly longer [5]. These findings were consistent with a study of 3568 patients undergoing thyroid surgery at a single institution demonstrated that the length of stay for octogenarians was significantly longer than the younger counterpart [15]. Grogan et al. had found that elderly (1.4 days) and superelderly (1.8 days) had significant increases in the hospital stay compared with the young (1.1 days) [4]. Therefore, there appeared to be a linear relationship between the age of patients and the length of hospital stay after surgery. However, similar relationships between age and hospital stay were also observed in other types of surgery such as cardiac or oncology [32]. These findings remained significant even after controlling for a number of clinical and demographic confounding factors.

### 4.4. Mortality

Direct comparison with other studies examining the rates of mortality in elderly patients is difficult since population groups and types of procedures differ. The data on thyroid surgery in elderly patients for mortality remains very limited (Table 2). In term of mortality, thyroidectomy is safe in elderly patients in experienced hands [3]. In studies with similar age spread, the 30-days mortality rates following thyroid surgery are between 0 to 0.8% [3–6, 10, 13, 15, 16]. Two deaths were recorded in the elderly group (0.87%), both due to advanced ATC and one death in the 50–60 years group (0.1%) caused by ischemic heart disease [3]. Since the mortality occurs rarely in thyroid surgery, both surgical- and non-surgical-related complications appear to be good end points for measuring the success of thyroid surgery in the elderly. Nevertheless, larger, prospective studies that include overall outcomes, long-term survival with mortality and quality of life for elderly patients with operative thyroid disease, are required.

### 4.5. Cost

Thyroid cancer increases in incidence and aggressiveness with age [33]. The elderly are the fastest growing segment of USA population. Reducing rates of rehospitalization would lower cost and improve quality of care [34]. Recently, rehospitalization rates have gained prominence in light of discourse about healthcare reform. There also have been numerous reports in the popular press suggesting that hospital readmission rates should be used as a measure of quality of care and be tied to physician and hospital reimbursement [35, 36]. Rohr showed that nearly one in five Medicare beneficiaries are hospitalized within 30 days of a hospital discharge, at a cost of $17.4 billion per year [37]. A large population-based study reported that patients 65 years and older had substantially longer hospitalizations than younger patients, and their in-hospital care was associated with substantially higher mean total costs [6]. For example, average LOS for thyroidectomy in a superelderly patient was more than a day longer than an elderly patient and more than twice that for a patient 18 to 44 years (3.7 days versus 1.7 days, resp.; *P* < 0.001). Average cost of thyroidectomy in a patient 80 years and older was $8,429, 39% more than the cost of the procedure in a patient 65 to 79 years, 55% more than a patient 45 to 64 years, and 59% more than a patient 18 to 44 years (*P* < 0.001). To our knowledge, the literature on rehospitalization and cost after thyroidectomy on elderly is very limited. Only one population-based study [10] and one SEER-database-based study [34] describe patterns of rehospitalization after thyroidectomy in elderly patients. Tuggle at al demonstrated that 4% readmission rate was caused by endocrine-related complications and another 4% were for the nonendocrine complications [34]. Seybt et al. also reported that higher rate of readmission in the elderly group (4.5%) as compared to the young patients (1.2%). However, this finding was not significant and none of the readmissions were attributable to a cause related to age as all were caused by hypocalcemia [10]. Furthermore, thyroidectomy has been shown to be safer in high-volume surgeon centers than in low-volume surgeon (one to thirty thyroidectomies per year) centers [14, 38]. Sosa et al. [14] reported the highest-volume surgeon had the lowest complication rate (2.4%; *P* < 0.01), adjusted length of stay (1.1 days; *P* < 0.001), and hospital charges ($2990 versus $3620 to $4420; *P* < 0.001) compared to all surgeon volume groups. However, the lowest-volume surgeons did the largest share of the thyroidectomies for all age groups. When combined with the fact that 50–80% of thyroid operations in the United States are done in low-volume centers, the results of these single-institution experiences are not generalizable to the typical thyroidectomy patient [39].

## 5. Quality of Life (QOL)

To our knowledge, literature that addresses QOL in patients with thyroid disease is very limited particularly for elderly patients. The lack of literature is surprising given that thyroid disease has been shown to have a significant impact on QOL [40]. Matsuyama et al. evaluated 85 patients with thyroid cancers and concluded that surgery for thyroid cancer increased the survival rate and promoted the quality of life of elderly patients if they could tolerate the procedure [20]. Others found that pneumonia and urinary tract infection had significant adverse effects on the QOL in elderly patients after thyroidectomy [41, 42]. A retrospective survey of 7000 cancer patients at M. D. Anderson Cancer Center, 518 of whom were thyroid cancer survivors, found that two-thirds (64.5%) of the thyroid cancer patients reported that their illness had significantly impacted their quality of life [43]. Surprisingly, when all other cancer survivors were asked the same question, only 32.1% reported that their illness had a significant impact. Shah et al. prospectively evaluated seventy-six patients with DTC and reported that patients with cancer experienced a greater drop in QOL during the first 6 months following surgery when compared with patients with benign disease (*P* < 0.03). However, patients treated with total thyroidectomy did not have a significantly different QOL than patients treated with hemithyroidectomy (*P* > 0.2) and they concluded that QOL should not be a factor in the decision-making process for the treatment of low-risk DTC [40]. A recent study from Germany also reported that thyroid surgery could be performed safely and without impairment of QOL, regardless of the extent of the surgery in patients with benign goiter [44].

## 6. Conclusion

Even with all the knowledge acquired today, the safety of thyroid surgery in elderly patients remains controversial. Although retrosternal goiter is a common surgical indication in the elderly and its prevalence might be more common than previously believed, the natural history of the disease in the elderly remains unknown and so it is difficult for surgeons to advise on elderly patients whether surgery is really beneficial in the long term. In well-selected patient cohort done by high-volume and experienced surgeons, no significant increase in surgical morbidity and mortality could be demonstrated. When dealing with elderly patients with surgical thyroid disease, careful patient selection and making referral to specialized high-volume center are necessary. Elderly patients with concomitant medical problems require to complete a thorough preoperative workup and assessment before undergoing any thyroid surgery. Any pulmonary, cardiac, or renal comorbidities should be optimized preoperatively. More data are needed in the future for analyses concerning indications for thyroid operations in aging population and the overall benefits, including long-term survival, quality of life, recurrence, and the effects of subsequent need for additional treatment of their thyroid disease. Larger prospective, population-based studies will probably provide better understanding and optimal approach to the elderly population in thyroid surgery.

## Figures and Tables

**Table 1:** A comparison of patient characteristics, definitions, indications, and procedures between different elderly surgical series.

Study/year	Design	Definition of “elderly” (years)	Number of patients (%)	Indications	Types of procedure
Bliss et al. [3] (1999)	Retrospective	(1) Elderly ≥75 (2) 61–74 (3) 50–60	(1) 221 (13.5) (2) 685 (42.1) (3) 725 (44.4)	(i) Compression symptoms	(i) Total thyroidectomy
				(ii) Risk of malignancy	(ii) Hemithyroidectomy
				(iii) Hyperparathyroidism	(iii) Total Lobectomy & subtotal thyroidectomy
				(iv)** Retrosternal goiter** ^∗^	(iv) Bilateral subtotal thyroidectomy
				(v) Toxic symptoms	(v) Enucleation of nodule
				(vi) Completion	(vi) Isthmectomy
				(vii) Others	(vii) Others

Passler et al. [16] (2002)	Retrospective	(1) Geriatric ≥ 75 (2) Younger < 75	(1) 55 (7.5) (2) 683 (92.5)	(i)** Benign** ^∗^ (ii) Suspected malignancy (iii) Malignancy (iv) Mechanical symptoms	(i) Radical—total/near-total thyroidectomy/hemithyroidectomy/neck dissection
					(ii) Nonradical—Enucleation/unilateral subtotal/bilateral thyroidectomy/hemithyroidectomy with contralateral subtotal resection

Lang and Lo [5] (2005)	Retrospective	(1) Elderly ≥ 70 (2) Young < 70	(1) 55 (18.5) (2) 242 (81.5)	(i) Compression symptoms	(i) Total thyroidectomy (ii) Near-total thyroidectomy
				(ii)** Thyrotoxicosis** ^∗^	
				(iii) **Recent enlargement** ^∗^	
				(iv) **Cosmesis**/**patient ** **request** ^∗^	
				(v) Suspicious FNAC	

Sosa et al. [6] (2008)	Cross sectional (population based)	(1) Superelderly ≥ 80	(1) 744 (3.3)^∗^	NA	(i)** Subtotal**/**total thyroidectomy** ^∗^
		(2) Elderly 65–79	(2) 4092 (17.9)^∗^		(ii)** Substernal thyroid** **e** **ctomy** ^∗^
		(3) 45–64	(3) 9959 (43.6)		
		(4) 18–44	(4) 8053 (35.2)		

Mekel et al. [15] (2009)	Retrospective	(1) Octogenarian ≥ 80 (2) 18–79		(i) Benign	(i) Lobectomy/partial thyroidectomy
			(1) 90 (2.5)	(ii) Malignancy	(ii) Total/subtotal thyroidectomy
			(2) 250 (randomly selected)	(iii) Microfollicular	
				(iv) Previous thyroid surgery	

Grogan et al. [4] (2012)	Prospective cohort (population based)	(1) Superelderly ≥ 80	(1) 199 (2.51)	NA	(i) Total thyroidectomy
		(2) Elderly 65–79	(2) 1322 (16.7)		(ii) Lobectomy
		(3) 16–64	(3) 6394 (80.8)		(iii) Completion
					(iv) With neck dissection
					(v) Substernal goite cervical approach
					(vi) Substernal goiter thoracic approach

Syebt et al. [10] (2012)	Prospective non-randomized	(1) Elderly > 65 (2) Young 21–35	(1) 44 (10.7) (2) 86 (20)	(i) Benign (ii) Malignancy	(i) Conventional thyroidectomy
					(ii) Minimally invasive open thyroidectomy
					(iii) Endoscopic thyroidectomy

^∗^Statistically significant (*P* < 0.01).

**Table 2:** A comparison of pathology, readmission rate, monetary cost, and hospital mortality in different elderly patients' series.

Study	Design	Pathology	Readmission	Cost	Mortality (within 30 days)
Bliss et al. [3] (1999)	Retrospective	(i)** MNG** ^∗^			
		(ii)** Carcinoma** ^∗^			
		(iii)** Adenoma** ^∗^			0.87% (elderly)
		(iv) Hashimato's	NA	NA	0.1% (61–74)
		(v) Single nodule			0.1% (50–60)
		(vi) Graves			
		(vii) Others			

Passler et al. [16] (2002)	Retrospective	(i) PTC			
		(ii) FTC			
		(iii) ATC			
		(iv) MTC	NA	NA	0%
		(v) MTC + DTC			
		(vi) Others			

Lang and Lo [5] (2005)	Retrospective	(i)** Functional (toxic) ** **MNG** ^∗^	NA	NA	0%
		(ii) Occult PTC			

Sosa et al. [6] (2008)	Cross sectional	(i)** Benign** ^∗^		^ #^(1)** $ 7084 (** **6653-7514** **)** ^∗^	(1) 0.8%
		(ii)** Adenoma and cyst** ^∗^	NA	^ #^(2)** $ 5917 ** **(5769-6066)** ^∗^	(2) 0.2%
		(iii)** Malignancy** ^∗^		^ #^(3)** $ 5263 (** **5182-5344** **)** ^∗^	(3) 0.1%
				^ #^(4)** $ 4905 ** **(4821-4990)** ^∗^	(4) 0%

Mekel et al. [15] (2009)	Retrospective	(i) Benign	(1) 1.1% for hypocalcaemia (2) 0%		
		(ii) Malignancy		NA	
		(iii) Incidental micropapillary			0%

Grogan et al. [4] (2012)	Prospective cohort	NA	NA	NA	0%

Syebt et al. [10] (2012)	Prospective non-randomized	(i) Benign	(1) 4.5%	NA	
		(ii) Malignancy	(2) 1.2%		0%

^∗^Statistically significant (*P* < 0.01).

^#^Adjusted for race, gender, hospital region, procedure, diagnosis, comorbidity, surgeon volume, household income, primary payer, and admission type in superelderly, elderly, 45-64 and 18-44 cohorts.

PTC: Papillary thyroid cancer, FTC: Follicular thyroid cancer, MTC: Medullary thyroid cancer, DTC: Differentiated thyroid cancer.

**Table 3:** A comparison of complications between different elderly surgical series.

Study/age group	Surgically related complications						Non-surgically related complications
	Temporary VC palsy	Permanent VC palsy	Temporary hypocalcemia	Permanent hypocalcemia	Hematoma	Wound infection
Bliss et al. [3]							
50–60	NA	1.4%	5%	0.2%	0.9%^#^	0.6%	
61–74		1.6%	5.2%	0.1%	1.2%^#^	0.4%	NA
Elderly		1.8%	3.9%	0.4%	0.9%^#^	0.4%	

Passler et al. [16]							
Geriatric	3.6%	1.05%	13.6%	2.3%	5.5%	1.8%	3.6%^*∧∧*^
Young	3.3%	0.26%	14.1%	5.4%	3.5%	0.4%	0.6%^*∧∧*^

Lang and Lo [5]							
Elderly	3.6%	0.9%	14.5%	1.8%	1.8%	0%	5.5%^*∧*^
Young	3.3%	0.6%	19%	1.2%	0.8%	0%	0.4%^*∧*^

Sosa et al. [6]							
Superelderly	**3.6**% ^∗^						**6.6**%^∗^
Elderly	**2.4**% ^∗^						**4.3**% ^∗^
45–64	**1.2**% ^∗^						**2.2**% ^∗^
18–44	**1.1**% ^∗^						**1.5**%^∗^

Mekel et al. [15]							
Elderly	**5.5**%^∗^						**18.9**% ^∗^
18–79	**2.5**%^∗^						**5.2**%^∗^

Grogan et al. [4]	(Overall complications)						
Superelderly	**5**%^∗^						
Elderly	**2.2**% ^∗^						
16–64	**1**% ^∗^						

Syebt et al. [10]							
Elderly	2.9%	0%	6.8%	0%			
Young	3.9%	0%	5.8%	0%	NA	NA	NA

^∗^Statistically significant (*P* < 0.01).

VC: vocal cord.

^#^ Included haematoma with evacuation.

^*∧*^Included pneumonia, cardiac complications, and perforated peptic ulcer.

^*∧∧*^Included respiratory distress, pneumonia, and paralysis of brachial plexus.
